# A Genetically Modified attenuated *Listeria* Vaccine Expressing HPV16 E7 Kill Tumor Cells in Direct and Antigen-Specific Manner

**DOI:** 10.3389/fcimb.2017.00279

**Published:** 2017-06-29

**Authors:** Yan Yan Jia, Wei Jun Tan, Fei Fei Duan, Zhi Ming Pan, Xiang Chen, Yue Lan Yin, Xin An Jiao

**Affiliations:** Jiangsu Key Laboratory of Zoonosis, Joint International Research Laboratory of Agriculture and Agri-Product Safety, Jiangsu Co-Innovation Center for Prevention and Control of Important Animal Infectious Disease and Zoonosis, Key Laboratory of Prevention and Control of Biological Hazard Factors (Animal Origin) for Agrifood Safety and Quality, Ministry of Agriculture of China, Yangzhou UniversityYangzhou, China

**Keywords:** attenuated *Listeria monocytogenes*, cervical cancer, HPV16 E7 oncoprotein, antitumor mechanism, direct tumoricidal effect, cell-mediated immunity

## Abstract

Attenuated *Listeria monocytogenes* (*L. monocytogenes*, LM) induces specific CD8^+^ and CD4^+^ T cell responses, and has been identified as a promising cancer vaccine vector. Cervical cancer is the third most common cancer in women worldwide, with human papillomavirus (HPV), particularly type 16, being the main etiological factor. The therapeutic HPV vaccines are urgently needed. The E7 protein of HPV is necessary for maintaining malignancy in tumor cells. Here, a genetically modified attenuated LM expressing HPV16 E7 protein was constructed. Intraperitoneal vaccination of LM4Δ*hly*::E7 significantly reduced tumor size and even resulted in complete regression of established tumors in a murine model of cervical cancer. We provided evidence that recombinant LM strains could enter the tumor tissue and induce non-specific tumor cell death, probably via activation of reactive oxygen species and increased intracellular Ca^2+^ levels. LM4Δ*hly*::E7 effectively triggered a strong antigen-specific cellular immunity in tumor-bearing mice, and elicited significant infiltration of T cells in the intratumoral milieu. In summary, these data showed LM4Δ*hly*::E7 to be effective in a cervical cancer model and LM4Δ*hly*::E7 induced an antitumor effect by antigen-specific cellular immune responses and direct killing of tumor cells, indicating a potential application against cervical cancer.

## Introduction

Cervical cancer is the third most common female cancer worldwide. About 530,000 new cases and 275,000 deaths are reported annually, with 85% and 88% of cases and deaths, respectively, occurring in developing countries (Ferlay et al., 2015; Torres-Poveda et al., 2016). Conventional therapeutic approaches for cervical cancer are based on chemotherapy and radiation therapy, but the 5-year survival of patients is dismal (Lee et al., 2013; Killock, 2015). Over 99% of cervical cancer is caused by persistent infection with human papillomavirus (HPV), particularly type 16. Currently, although two prophylactic virus-like particle-based vaccines are available, they have no effect on existing HPV-associated diseases. Furthermore, HPV infections remain extremely common. Thus, there remains an urgent need to develop therapeutic HPV vaccines (Depuydt et al., 2016; Stanley, 2016). Cancer immunotherapy has emerged as an alternative, innovative approach that can induce the persistent cell-mediated immunity necessary to retard tumor growth. So far, HPV16 E6 and E7, which are constitutively expressed in cervical cancer cells, seem to be the most promising target genes for therapeutic vaccines against cervical cancer (Grabowska et al., 2015). Several types of therapeutic vaccines against HPV-associated cancer have been developed and tested in preclinical trials, including protein, live vector, nucleic acid, and cell-based vaccines. Live bacterial vector-based therapeutic HPV vaccines are one of the most promising vector vaccines because of highly immunogenic and convenient. Nowadays, *Listeria monocytogenes* (*L. monocytogenes*, LM), *Lactobacillus lactis*, and *salmonella* are the most common bacteria-based vectors for tumor immunotherapy (Yang A. et al., 2016). Among the intracellular bacteria, *Listeria* has been recognized as a promising vector because of its unique immunologic properties (Rothman and Paterson, 2013).

*L. monocytogenes* is a gram-positive, facultative intracellular bacterium. Once inside cells, the bacterium produces specific virulence factors which lyse the vaculolar membrane and allow escape into the cytoplasm. *L. monocytogenes* in the cytosol is capable of inducing CD8^+^ and CD4^+^ T cell responses via MHC class I and MHC class II pathways (Pillich et al., 2015). LM also induces a decrease in regulatory T cells (Tregs) in tumor tissue, thus favoring immune responses that can kill tumor cells (Chen et al., 2014). These characteristics make attenuated LM a promising candidate cancer vaccine vector (Wood and Paterson, 2014; Wan et al., 2015).

Previous studies have reported that attenuated LM was applied to deliver the HPV E7 gene via a plasmid-based complementation system, but there existed a high risk of plasmid loss and persistence of antibiotic resistance genes in clinical trials (Peng et al., 2004; Chen et al., 2014). Additionally, Wallecha demonstrated that detoxified listeriolysin (LLO) as a fusion partner, or mixed with E7 antigen which were expressed by *E. coli* BL21(DE3) could augment specific immune responses and significant reduction of tumor growth (Wallecha et al., 2013), but the protein-based vaccine was expensive and low immunogenicity. In the present study, a genetically modified attenuated recombinant LM4Δ*hly*::E7 strain was constructed to express and secrete the LLO-E7 fusion protein. The study presented here demonstrates that this recombinant (r) LM vaccine is safe and effectively retards tumor growth in a mouse model of cervical cancer. Furthermore, we provide evidence that the antitumor mechanism is due to induction of specific cytotoxic T lymphocyte (CTL) responses and direct killing of tumor cells.

## Materials and methods

### Peptides, bacteria, and plasmids

HPV16 E7_49–57_ (RAHYNIVTF) peptide for the H-2D^b^ restricted epitope was synthesized by Beijing Scilight Biotechnology, LLC (Beijing, China). Virulent *L. monocytogenes* Yangzhou strain yzuLM4 (serotype 1/2a) was isolated and maintained at the Jiangsu Key Laboratory of Zoonosis, which was cultured in a Biosafety Level 2 laboratory. Shuttle vector pKSV7 was kindly provided by Dr. Guoqiang Zhu (Yangzhou University, Jiangsu). Plasmid pNF8, containing the full-length *gfp-mut1* gene was a gift from Prof. Nicolas Fortin, eau (Laboratoire de Bactériologie-Virologie, France).

### Mice and cell lines

C57BL/6 mice (7–8 weeks old) were purchased from the Comparative Medical Center of Yangzhou University. Animals were housed and used in accordance with the protocols approved by the Experimental Animal Center Institutional Committee of Yangzhou University. The TC-1 cell line, C57BL/6 lung tumor epithelial cells immortalized by HPV16 E6/E7 and transformed with the c-Ha-ras oncogene, was purchased from the Tumor Cell Center of the Chinese Academy of Medical Sciences (Beijing, China). RAW264.7 murine macrophage-like cells were maintained in our laboratory. Cells were cultured in RPMI 1640, supplemented with 10% fetal calf serum, 1 mmol/L sodium pyruvate, 100 U/mL penicillin, and 100 μg/mL streptomycin in a 37°C incubator with 5% CO_2_.

### Construction of recombinant *L. monocytogenes* and antigen expression

The upstream fragment *hly*a and downstream fragment of *hly*b were amplified from the LM4 genome using the following primers: *hly*a (F) 5′-CCGGTACCAGGTTTGTTGTGTCAGGTAGAGC-3′, *hly*a (R) 5′-GTAGGTGTATCTCCATGTGAAATTGAATTTTCTTTAT-3′; *hly*b (F) 5′-TGTTCTCAGAAACCACCAATCGAAAAGAAACACG-3′, *hly*b (R) 5′-GGGTCTAGAACTTGAGATATATGCAGGAGG-3′; the *Bam*HI and *Xho*I sequences are underlined. HPV16 E7 gene was amplified from pcDNA3.1-E7 using the following primers: E7 (F) 5′-ATAAAGAAAATTCAATTTCACATGGAGATACACCTAC-3′, E7 (R) 5′-CGTGTTTCTTTTCGATTGGTGGTTTCTGAGAACA-3′. *hly*a, *hly*b and E7 were spliced by overlap extension PCR with the primers *hly*a (F) and *hly*b (R). The fusion fragment aEb was cloned into the shuttle vector pKSV7 with restriction enzyme sites *Bam*HI and *Xho*I. The resulting plasmid pKSV7-aEb was electroporated into electrocompetent LM4 cells. The E7 gene was cloned in-frame with the *hly* open reading frame and inserted downstream of the *hly* signal sequence by allelic exchange, and a 42 bp region was deleted from the N-terminal Pro-Glu-Ser-Thr (PEST)-like sequence of the *hly* gene. The recombinant plasmid- and antibiotic-resistance gene-free strain LM4Δ*hly*::E7 was constructed to express and secrete LLO-E7 fusion protein, retaining a significant degree of attenuation. An irrelevant *Listeria* construct, LM4Δ*hly*::*esat-6* (LM control), which was based on the same method of construction, was included to account for the antigen-independent effects of *Listeria* on the immune system. Supernatants from the recombinant strain cultures were concentrated by trichloroacetic acid (TCA), and detected by Western blot using a monoclonal antibody against HPV16 E7 protein.

### Hemolysis test

LM4 and LM4Δ*hly*::E7 were cultured overnight in brain-heart infusion (BHI) medium. The culture supernatants were collected by centrifugation, and the same *OD*_600_ value was adjusted using PBS buffer. Seventy microliters of each supernatant were added to a 96-well plate, and then two-fold serial dilution was performed. Subsequently, 30 μL of 1% sheep erythrocytes were added, and hemolysis was observed after 1 h at 37°C. Hemolysis titers were expressed as the dilution of the supernatant that lysed 50% of total sheep erythrocytes.

### Macrophage invasion study *in vitro*

Firstly, pNF8 was electroporated into electrocompetent LM4 and LM4Δ*hly*::E7 cells, resulting in construction of LM4(pNF8) and LM4Δ*hly*::E7(pNF8). Subsequently, RAW264.7 cells cultured in 24-well plates were infected with LM4(pNF8) or LM4Δ*hly*::E7(pNF8) at a multiplicity of infection of 1:20 for 1 h. The cells were incubated in medium containing 50 μg/mL of gentamicin for 1 h at 37°C to eliminate extracellular bacteria. The cells were fixed and stained with phalloidin (Invitrogen), which stains filamentous actin, by taking samples at 1 and 5 h. Intracellular bacteria were monitored by confocal-transmission electron microscopy.

### Tumor model and immunization

To establish the tumor model, 2 × 10^5^ TC-1 cells were subcutaneously injected into the left flank of C57BL/6 mice. After 1 week, tumor-bearing mice (tumor diameter reached 5 mm) were intraperitoneally administered with 0.1 50% lethal dose (LD_50_) of LM4Δ*hly*::E7, LM4Δ*hly*::*esat-6* or PBS on days 7 and 14 after tumor cell injection. Tumors were monitored, and measured at the longest (L) and widest (W) points with calipers. Tumor volume was calculated as L × W^2^ × π/6. Experiments were initiated in the tumor-bearing mice to analyze the anti-tumor immune response.

### Kinetics and distribution of Lm4δ*hly*::E7 in *in vivo* infection model

The tumor-bearing mice were intraperitoneally injected with LM4Δ*hly*::E7 on days 0, 7, and 14. Spleens, livers and tumors were homogenized on days 1 and 3 after each immunization. Bacterial numbers were determined by plating the cell suspensions on BHI agar.

### *In vivo* cytotoxicity assay

A standard *in vivo* killing assay was performed as previously described (Coles et al., 2002). In brief, splenocytes were pooled from naive C57BL/6 mice and divided into two groups. One was labeled with 2.5 μM carboxyfluorescein succinimidyl ester (CFSE^high^; Molecular Probes, Invitrogen, Carlsbad, CA) buffer, another was labeled with 0.25 μM CFSE (CFSE^low^). CFSE^high^ cells were pulsed with the E7 peptide. CFSE^high^ and CFSE^low^ cells were mixed at a 1:1 ratio and injected intravenously into C57BL/6 mice at 7 days post-immunization. Fifteen hours later, spleens were harvested and processed into single cell suspensions. The splenocytes were analyzed by flow cytometry according to fluorescence density, and the specific lysis ratio was calculated with the following formula:100 – (100 × (% CFSE^high^ immunized/%CFSE^low^ immunized)/(% CFSE^high^ control/% CFSE^low^ control)).

### ELISPOT assay

The ELISPOT assay was performed as previously described (Jia et al., 2012). Briefly, splenocytes were seeded on an anti-murine interferon-gamma (IFN-γ)/interleukin (IL)-4-coated ELISPOT plate (BD Biosciences Pharmingen, San Diego, CA) at a density of 2 × 10^5^/well. Cells were stimulated in triplicate with E7_49–57_ peptide (10 μg/mL), with ConA (5 μg/mL, Sigma) as a positive control or complete medium as a negative control, and incubated at 37°C for 48 h. Samples were then processed according to the manufacturer's instructions (BD Biosciences Pharmingen, San Diego, CA). The IFN-γ/IL-4-reactive spots were quantified by an automated ELISPOT Bioreader 5000 (ImmunoBioSystem, The Colony, TX).

### Flow cytometric analysis of CD4^+^ and CD8^+^ T cells

On day 7 after the second immunization, tumors were excised, and cut into 1–2 mm pieces using a sterile razor blade and digested with PBS buffer containing 2 mg/mL collagenase type I and 12 U/mL DNase (Invitrogen, Carlsbad, CA, USA) for 2 h at 37°C with agitation. Single cell suspensions were pooled through a nylon mesh.

Cells were stained with anti-CD3-fluorescein isothiocyanate (FITC) (clone 145-2C11; BD Pharmingen, San Diego, CA) anti-CD4-phycoerythrin (PE) (clone GK1.5; BD Pharmingen) and anti-CD8-allophycocyanin (APC) (clone 53-6.7; BD Pharmingen) monoclonal antibodies and then analyzed by flow cytometry.

### Immunohistochemical analysis of the tumor tissue

Tumor specimens on day 7 after the second immunization were fixed in 13% neutral buffered formalin and embedded in paraffin. Sections were cut at 5 μm thick, and incubated with anti-mCD8 (clone 53-6.7; R&D Systems, Minneapolis, MN) or anti-mCD4 (clone GK1.5; R&D Systems) antibody overnight at 4°C. The anti-mCD8 or anti-mCD4 antibody was visualized with a HRP-DAB Cell and Tissue Staining Kit (R&D Systems).

### Annexin V-FITC/PI staining

Tumor cell suspensions were obtained as described above. The cells were stained with FITC-labeled Annexin V (BD Pharmingen) and propidium iodide (PI; BD Pharmingen) for 15 min in the dark at room temperature. Subsequently, the rate of cell apoptosis and cell death were evaluated quantitatively by flow cytometry.

### Measurement of reactive oxygen species (ROS)

The generation of intracellular ROS was measured using a dichloro-dihydro-fluorescein diacetate (DCFH-DA) dye assay. DCFH-DA penetrates the cells and is hydrolyzed to DCFH by non-specific esterases. In the presence of cellular oxidizing agents, DCFH is rapidly oxidized to the highly fluorescent compound dichlorofluorescein (DCF), which is trapped inside the cells. Therefore, the fluorescence intensity is proportional to the amount of ROS generated in the cells. In brief, tumor cell suspensions were obtained as described above. The cells were stained with DCFH-DA (10 μM, Beyotime Institute of Biotechnology, Beijing) at 37°C for 20 min, and then washed to remove extracellular DCFH-DA. Changes in DCF fluorescence were assayed by flow cytometry.

### Measurement of intracellular calcium ion (Ca^2+^) concentration

Changes in intracellular Ca^2+^ levels were detected by Fluo-3 acetoxymethyl ester (Fluo-3 AM, Molecular probe Inc. USA). Intracellular Fluo-3 AM is deacetylated by esterases to yield Fluo-3, which is trapped inside the cells. Upon Ca^2+^ binding, Fluo-3 exhibits strong fluorescence. Briefly, the cells isolated from tumor tissue were incubated with 5 μM Fluo-3 AM at 37°C for 20 min, and then washed with PBS buffer to remove extracellular Fluo-3 AM. The fluorescence intensity of Fluo-3 was analyzed by flow cytometry.

### Statistical analysis

Statistical analysis for *in vitro* and *in vivo* experiments was carried out using GraphPad Prism Software (GraphPad Software Inc., La Jolla, CA). Student's *t*-test and one-way ANOVA were used for analysis of the comparisons between the groups. Statistical significance was established as *P* < 0.05 (^*^P < 0.05, ^**^P < 0.01, ^***^P < 0.001).

## Results

### Construction of LM4Δ*hly*::E7 expressing and secreting LLO-E7 fusion protein

**Figure 1:**
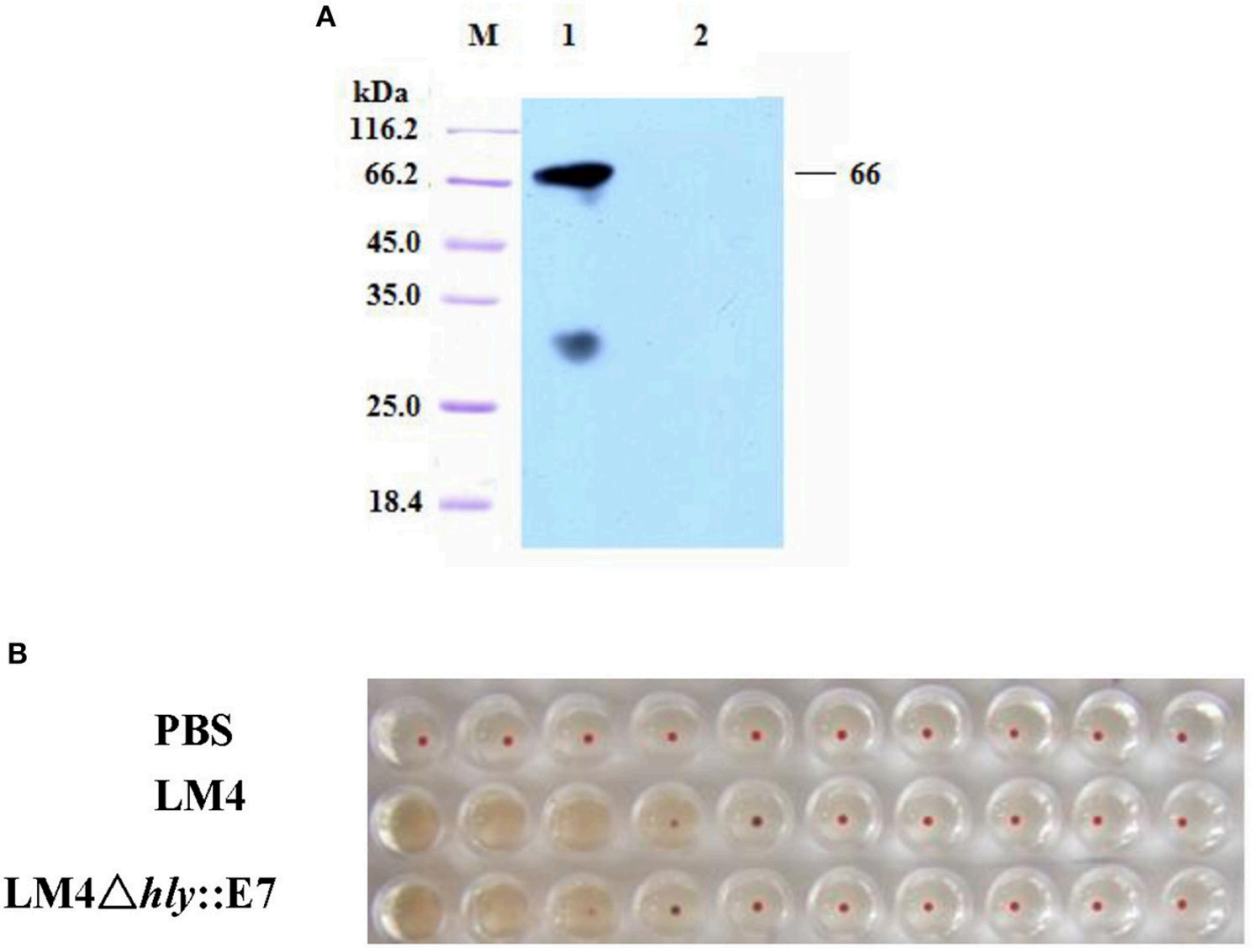
Characterization of the LM4Δ*hly*::E7 strain. **(A)** Western blot analysis of proteins secreted by LM4Δ*hly*::E7. The culture supernatant of LM4Δ*hly*::E7 was precipitated by TCA and secretory proteins were analyzed using a monoclonal antibody against HPV16 E7 by Western blot. M, protein marker; Lane 1, LM4Δ*hly*::E7; Lane 2, wtLM4. **(B)** Hemolytic activity of the recombinant strain was detected. The culture supernatants of LM4Δ*hly*::E7 and wtLM4 were two-fold serially diluted, 1% sheep blood cells were added and incubated at 37°C for 1 h.

LM4Δ*hly*::E7, which expresses and secretes the LLO-E7 fusion protein, was constructed by homologous recombination. In C57BL/6 mice, LM4Δ*hly*::E7 was highly attenuated and exhibited a LD_50_ of 3.863 × 10^9^ colony forming units (CFU) compared with the LD_50_ of wild type (wt) LM4, 5 × 10^4^ CFU. The LLO-E7 fusion protein secreted by LM4Δ*hly*::E7 was detected in Western blotting assay, which is 67 kDa in size (Figure 1A). Moreover, hemolytic activity of the fusion protein was tested by hemolysis assay, and its hemolysis titers reached 2^4^, slightly lower than that of the parent strain (Figure 1B).

### LM4Δ*hly*::E7 can infect and replicate in macrophages

**Figure 2:**
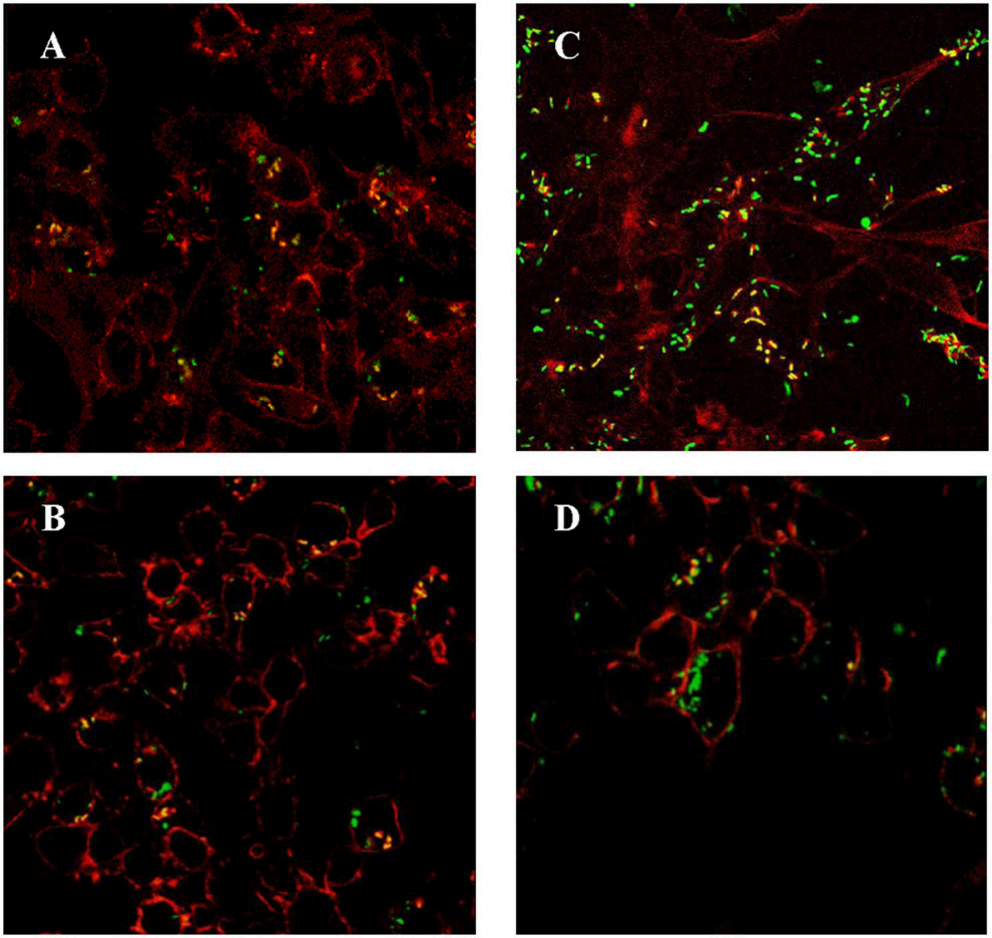
RAW264.7 cells infected with rLMs. LM4Δ*hly*::E7(pNF8) **(A)** and LM4(pNF8) **(B)** for 1 h, or LM4Δ*hly*::E7(pNF8) **(C)**, and LM4(pNF8) **(D)** for 5 h. Red, cytoskeleton; green, LM4Δ*hly*::E7(pNF8) bacilli or LM4(pNF8) bacilli; yellow in merge image. Images were taken with a confocal laser scanning microscope (Original magnification × 630).

LM4(pNF8) and LM4Δ*hly*::E7(pNF8) expressing green fluorescent protein were constructed and used to infect RAW264.7 cells, following which the multiplicity of these LM strains in RAW264.7 cells was determined. As shown in Figures 2A,B and S1, there were only a few bacteria infecting any given cell after 1 h incubation. However, after 5 h, the amounts of LM4(pNF8), and LM4Δ*hly*::E7(pNF8) were increased (Figures 2C,D and S1).

### Infection kinetics in organs from tumor-bearing mice immunized with LM4Δ*hly*::E7

**Figure 3:**
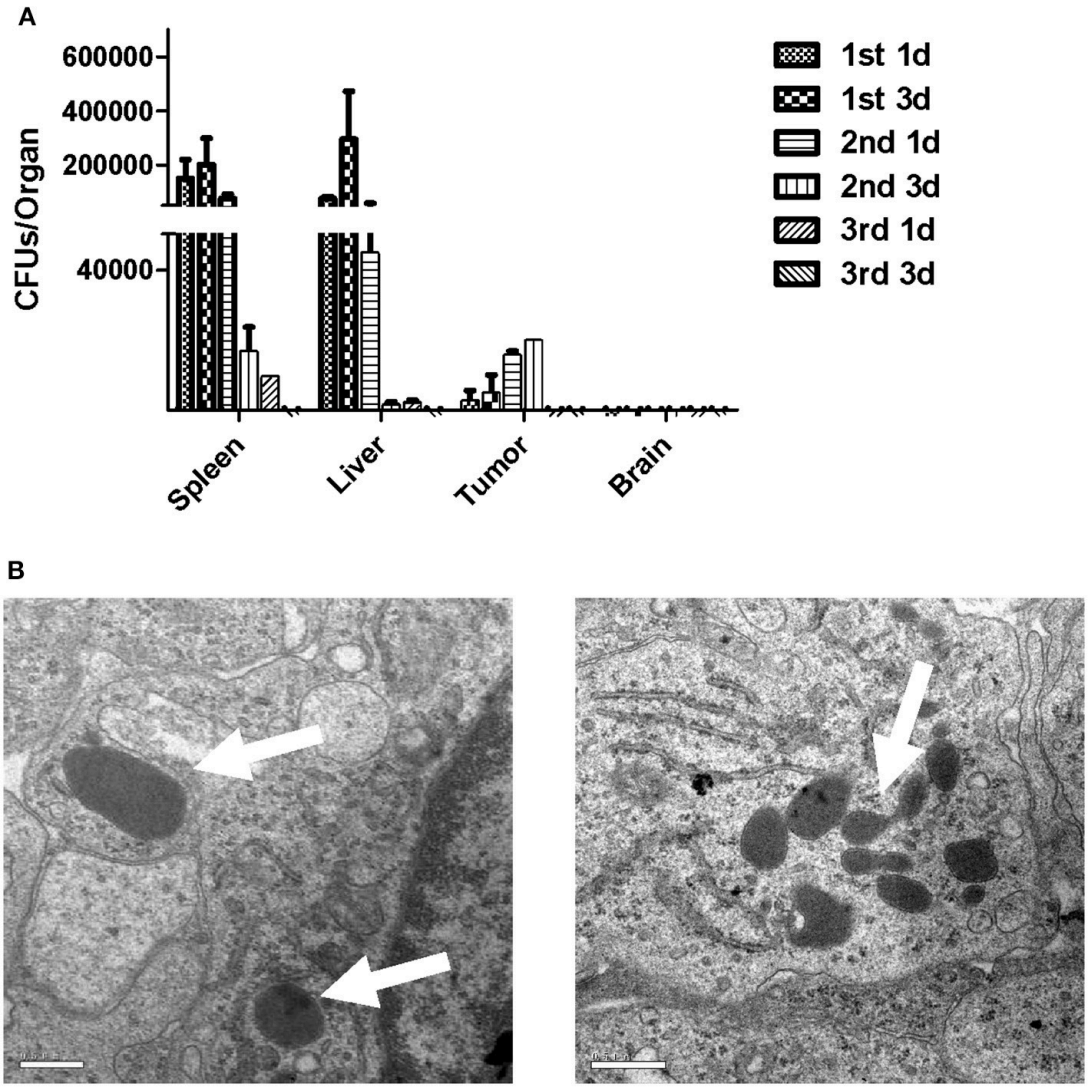
Infection kinetics in organs of mice immunized with LM4Δhly::E7. Groups of five tumor-bearing mice were intraperitoneally immunized with LM4Δ*hly*::E7 on days 0, 7, and 14. Spleens, livers, brains, and tumors were extracted and homogenized on 1 and 3 days after each immunization. Homogenates were diluted with PBS buffer and plated on BHI agar to determine bacterial numbers. **(A)** CFUs/organ was calculated on 1 and 3 days after each immunization, and infection kinetics in the organs are shown. **(B)** LM4Δ*hly*::E7 bacilli in the tumors were observed by electron microscopy. Scale bar 0.5 μm, white arrows, LM4Δ*hly*::E7 bacilli. Data are expressed as the mean and SD values from three independent experiments.

To determine the infection kinetics in different organs of tumor-bearing mice immunized with LM4Δ*hly*::E7, spleens, livers and brains were harvested, along with tumors from the immunized mice at different time-points. Tissues were homogenized and CFU/organ was calculated. The results showed that the bacterial numbers in spleen and liver peaked on the 3rd day after the first immunization, and gradually decreased if further immunization doses were administered. As expected, LM was not found in brain homogenates. LM4Δ*hly*::E7 was quantified in tumor tissues on the 1st day after the first immunization, and bacterial numbers started to increase, peaking at day 3 after the second immunization (Figure 3A). Using electron microscopy, numerous LM4Δ*hly*::E7 bacilli were observed in the tumors in Figure 3B.

### Vaccination with LM4Δ*hly*::E7 leads to stasis in tumor growth

**Figure 4:**
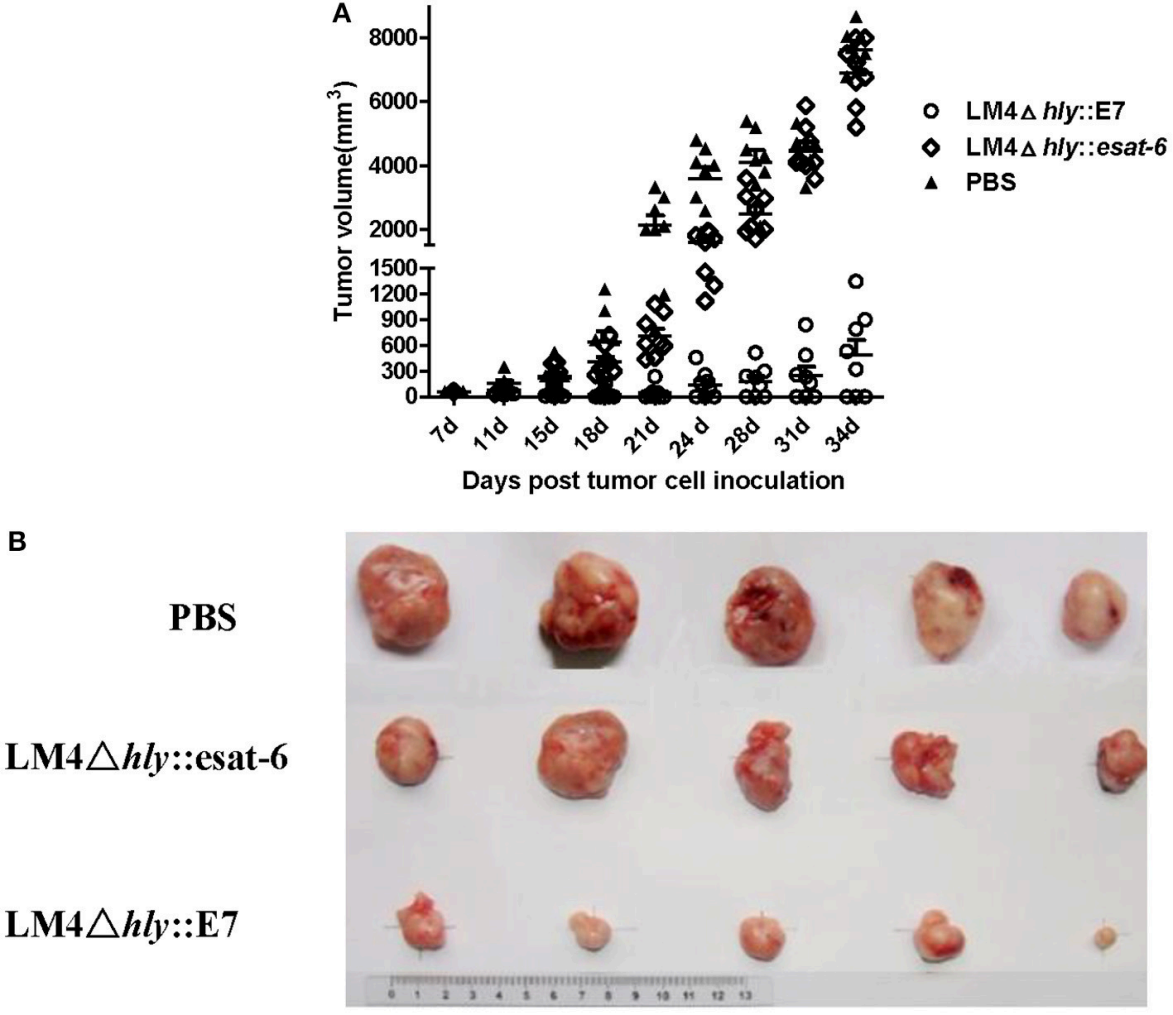
Tumor regression in tumor-bearing mice treated with rLMs. Groups of eight mice were subcutaneously injected with 2 × 10^5^ TC-1 cells. When the diameter of the tumor was 5 mm, mice were intraperitoneally immunized with LM4Δ*hly*::E7, LM4Δ*hly*::*esat-6* or PBS buffer. One week later, mice were given a booster immunization, and the tumors were monitored. **(A)** Tumor volume in each mouse in the different treatment groups was determined up to 34 days post tumor inoculation, ^**^ *P* < 0.01 vs. the LMΔ*hly*::*esat-6* and PBS groups by Student's *t*-test. **(B)** Representative tumors are shown. The data are representative of three independent experiments.

To assess the antitumor efficacy of LM4Δ*hly*::E7, tumor-bearing mice were intraperitoneally immunized twice with LM4Δ*hly*::E7, LM4Δ*hly*::*esat-6* or PBS buffer at weekly intervals. The tumor volume was measured twice weekly. As shown in Figures 4A,B, LM41hly::E7 significantly reduced tumor sizes and slowed the growth of tumors compared to the LM4Δ*hly*::*esat-6* and PBS groups. Moreover, three of eight mice in the LM4Δ*hly*::E7-immunized group demonstrated complete tumor regression.

### LM4Δ*hly*::E7 elicits strong E7-specific cellular immunity in tumor-bearing mice

**Figure 5:**
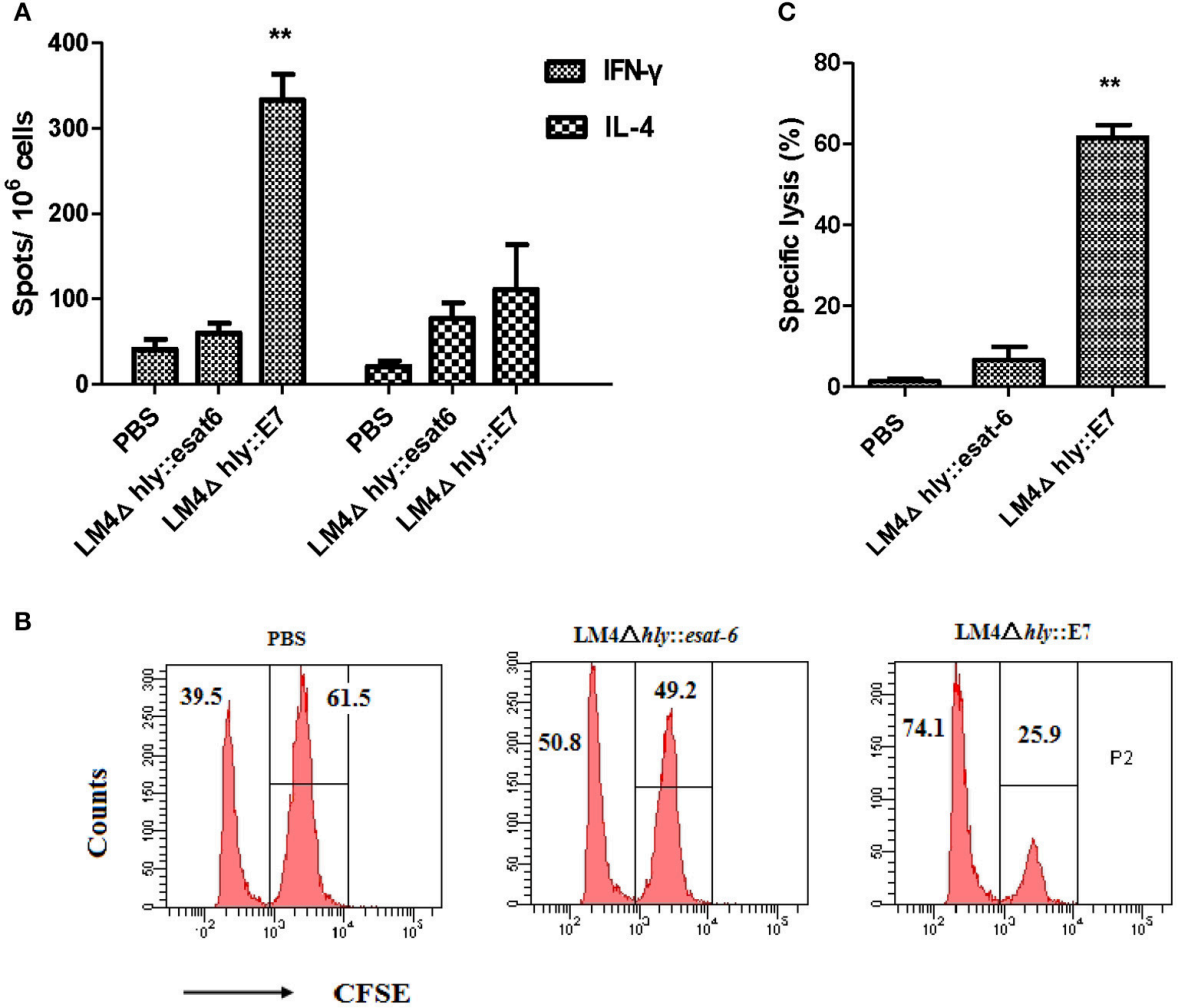
Cellular responses elicited by LM4Δhly::E7 in tumor-bearing mice. Groups of five mice were subcutaneously injected with 2 × 10^5^ TC-1 cells on day 0, and were intraperitoneally immunized with LM4Δ*hly*::E7, LM4Δ*hly*::*esat-6* or PBS buffer on days 7 and 14. On day 21, E7-specific cellular immunity was analyzed in immunized mice. **(A)** The number of IFN-γ- and IL-4-secreting cells in harvested splenocytes, assessed by ELISPOT assay. **(B)** Data representative of the E7-specific CTL response *in vivo*. **(C)** Specific lysis ratio by CTLs in different groups. Data are expressed as the mean and SD values from three independent experiments. ^**^P < 0.01 vs. the LMΔ*hly*::*esat-6* and PBS groups by Student's *t*-test.

In order to investigate the cellular immune responses induced by LM4Δ*hly*::E7 in tumor-bearing mice, the numbers of IFN-γ- and IL-4-secreting cells in splenocytes harvested 7 days after the second immunization were determined using an ELISPOT assay. As shown in Figure 5A, immunization with LM4Δ*hly*::E7 significantly increased the amount of E7-specific IFN-γ-secreting cells as compared with the control LM4Δ*hly*::*esat-6* and PBS groups (^**^P < 0.01). Also, the number of IFN-γ-secreting cells were significantly expanded compared with IL-4-secreting cells in the LM4Δ*hly*::E7-immunized group (^**^P < 0.01), which demonstrated that the immune response induced by LM4Δ*hly*::E7 is skewed toward a Th1 type response in tumor-bearing mice.

CTL activity *in vivo* was determined using a CFSE-based assay. As shown in Figures 5B,C, tumor-bearing mice immunized with LM4Δ*hly*::E7 generated a robust cytotoxic response with an E7-specific lysis ratio of 61.62 ± 6.2%, which was significantly higher than the control groups (^**^P < 0.01).

### LM4Δ*hly*::E7 induces the highest ratio of T cell subpopulations in tumors

**Figure 6:**
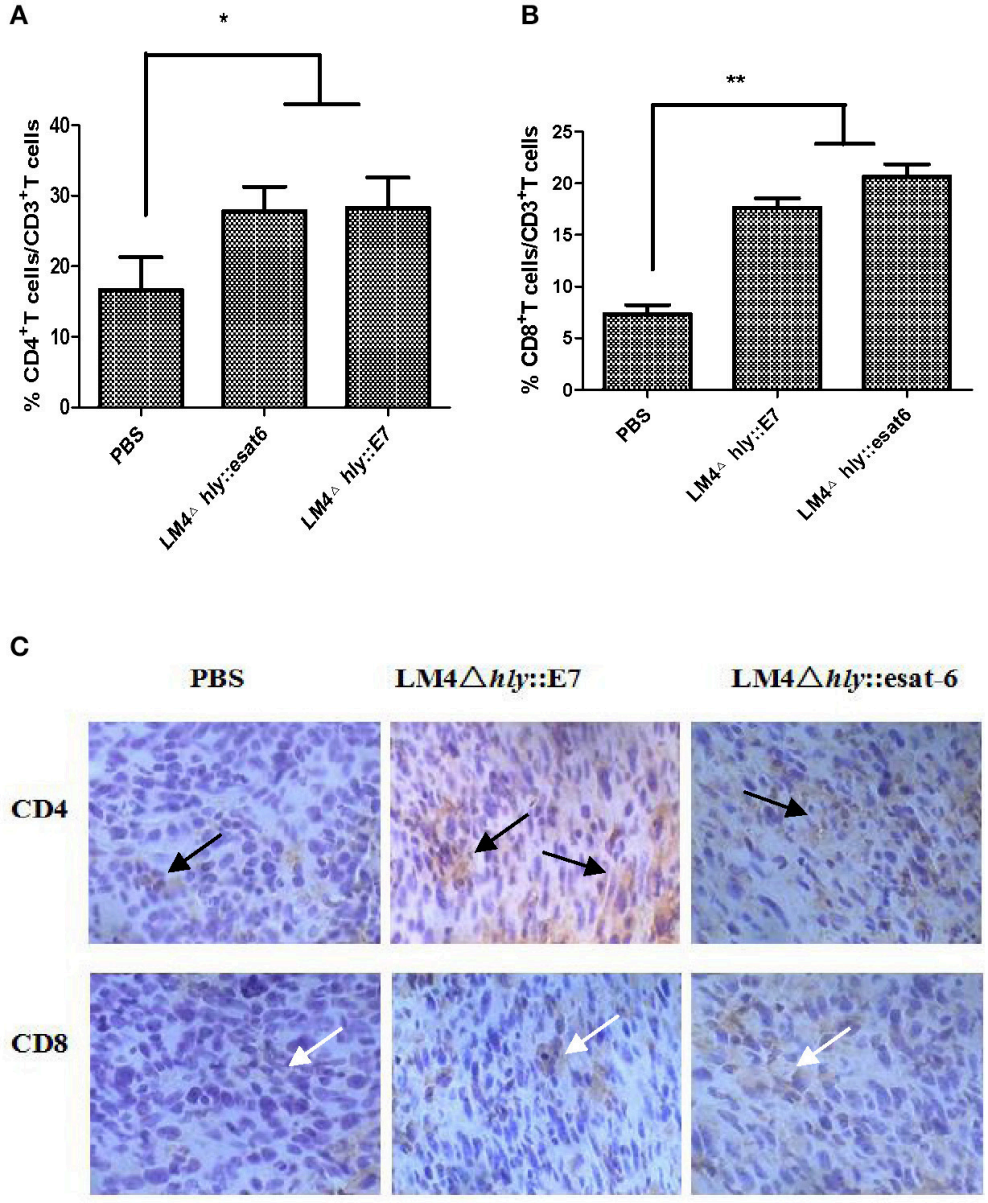
Characterization of CD4^+^ and CD8^+^ T cells in tumor tissues after immunotherapy. **(A,B)** Tumor cells were isolated 7 days after the final vaccination. Cells were then stained with anti-CD3-FITC, anti-CD4-PE, and anti-CD8-APC monoclonal antibodies, and analyzed by flow cytometry. **(C)** CD4^+^ and CD8^+^ T cells in tumor tissues were analyzed by immunohistochemistry (Orignal magnification ×200), black arrows, CD4^+^ T cells (brown granules), white arrows, CD8^+^ T cells (brown granules). Images are representative in the immunized groups and the PBS group. Data are expressed as the mean and SD values from three independent experiments. ^*^P < 0.05; ^**^P < 0.01, Student's *t*-test.

By comparing the percentages of T cell subpopulations isolated from tumor-bearing mice in the different experimental groups, we observed by flow cytometry that more CD4^+^ and CD8^+^ T cells infiltrated into the tumor site in the LM4Δ*hly*::E7 and LM4Δ*hly*::*esat-6* groups compared with the PBS group (Figures 6A,B). A similar result was found in the immunohistochemistry assay: high positive staining levels of CD4^+^ and CD8^+^ T cells were observed in the rLM-immunized groups (Figure 6C).

### Recombinant *Listeria* strains induce tumor cell death and cell apoptosis

**Figure 7:**
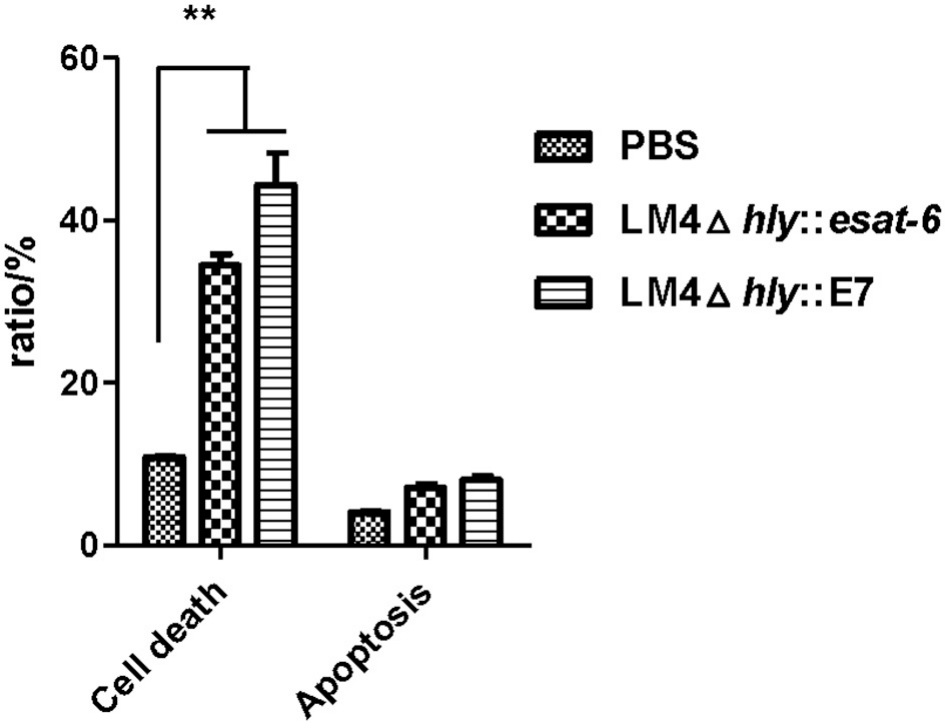
Cell death and apoptosis in the tumors of tumor-bearing mice treated with rLM. Tumors were excised 7 days after the final vaccination, and processed into single cell suspensions. Cells were then stained with Annexin-V-FITC and PI, and cell apoptosis and cell death were analyzed by flow cytometry. The rates of cell death were calculated. Data are expressed as the mean and SD values from three independent experiments. ^**^P < 0.01, Student's *t*-test.

Next, we investigated the changes of cell death in the tumor tissues. Tumor tissues from tumor-bearing mice treated with rLM were extracted and processed into single cell suspensions. By Annexin-V-FITC and PI staining, our results showed that LM4Δ*hly*::E7 and LM4Δ*hly*::*esat-6* could induce tumor cell death, which were significantly different from the PBS group (^**^P < 0.01). However, there were no significant differences between the two rLM-immunized groups (Figure 7).

### Recombinant *Listeria* strains induce higher intracellular ROS and Ca^2+^ levels in tumor cells

**Figure 8:**
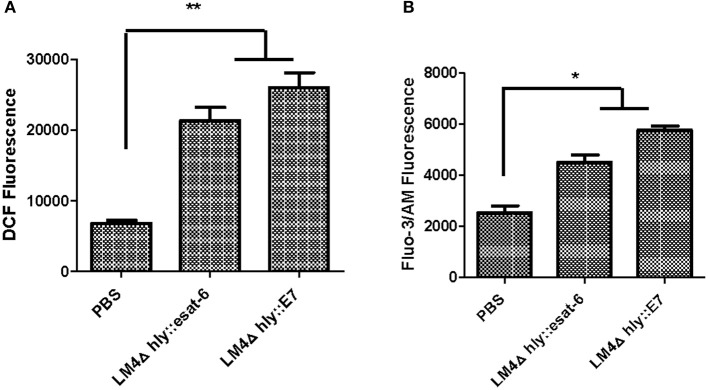
The level of intracellular ROS and Ca^2+^ in tumor cells from rLM-treated mice. Tumors were excised 7 days after the final vaccination, and processed into single cell suspensions. The cells were then stained with DCFH-DA dye assay or Fluo-3 AM assay, and the intracellular ROS **(A)** and Ca^2+^ levels **(B)** were determined by flow cytometry. Data are expressed as the mean and SD values from three independent experiments. ^*^P < 0.05; ^**^P < 0.01, Student's *t*-test.

Intracellular ROS assays in tumor cells indicated that LM4Δ*hly*::E7 and LM4Δ*hly*::*esat-6* could induce the generation of intracellular ROS compared with the PBS group (Figure 8A). Furthermore, the results revealed that rLM strains also induced elevations in intracellular Ca^2+^ concentration (Figure 8B).

## Discussion

Cervical cancer is a serious health concern, and is due to persistent infection with high-risk subtypes of HPV, including types 16, 18, 31, and 45 (Yuji and Nakada, 2016). Preventive vaccines for cervical cancer do not exert therapeutic effects on pre-existing HPV infections and HPV-associated lesions, and thus recent advances in immunotherapy treatment of cervical cancer have gained much attention lately (Yang M. C. et al., 2016). These therapeutic vaccines aim to generate cellular immune responses to tumor cells. *L. monocytogenes* is capable of escaping phagocytosis and inducing a strong cellular immune response (Cory and Chu, 2014). Studies have indicated that attenuated LM was able to induce antitumor immunity against a number of tumor associated antigens such as hepatic cancer stem cell biomarker CD24 and malignant melanoma MIA gene (Qian et al., 2012; Yang et al., 2014). Furthermore, fusion of the antigen (for example, the epidermal growth factor receptor family of tyrosine kinases, HER-2/neu; prostate-specific antigen, PSA and so on) to dtLLO or ActA (encoded by the *act*A gene) and secretion by *L. monocytogenes* was found to significantly enhance anti-tumor immune responses in comparison to an LM vector that secreted only the tumor antigen (Singh et al., 2005; Shahabi et al., 2008). In the present study, a new strategy involving genetic modification of an attenuated LM strain to express and secrete LLO-E7 fusion protein has been developed. While established tumors (tumor diameter>5 mm) are known to reside in a highly immunosuppressive microenvironment, LM4Δ*hly*::E7 could lead to the eradiction of the tumors by the end of the experiment.

Plasmid based complementation system was always applied as a method of constructing recombinant LM. However, plasmid retention often requires antibiotic resistance markers, the presence of which has been discouraged in clinical applications by the Food and Drug Administration (FDA) (Azizoglu et al., 2014). Perviously, studies reported that LLO-E7 fusion protein resulted in reductions in tumor volume (Wallecha et al., 2013). Subunit vaccines, however, suffer from low immunogenicity and most are presented through the MHC class II pathway which activates the production of antibodies instead of generating a CTL response. So HPV16 E7 was integrated into the downstream of *hly* signal sequence within LM genome by homologous exchange in this study, and LLO-E7 fusion protein stably secreted by *Listeria*. Although expression quantity of foreign antigen in this way is lower than that of plasmid retention system, the recombinant strain LM4Δ*hly*::E7 here led to the long-term regression of established tumors in a transplantable tumor model. These results indicated that the recombinant strain with this construction method was a more efficient delivery vector.

The role of therapeutic cancer vaccines is mainly to induce activation of antigen-specific CD8^+^ T cells, which can release proinflammatory cytokines and cytolytic agents to attack and kill tumor cells (Shahabi et al., 2008). Our results showed that T cells from immunized mice were able to recognize and lyse peptide-pulsed target cells *in vitro*, and that LM4Δ*hly*::E7 induced robust and durable E7-specific CTL responses in tumor-bearing mice. IFN-γ is a crucial cytokine in Th1 cell-mediated immunity against tumor cell proliferation (Mandai et al., 2016). The number of IFN-γ-producing T lymphocytes and the level of IFN-γ secretion in the LM4Δ*hly*::E7 immunized group were significantly higher than control groups. The strong cell-mediated response triggered by LM4Δ*hly*::E7 in a transplantable tumor model was effective for antitumor efficacy.

Interestingly, our results showed that intraperitoneally injected rLM strains entered the tumor tissues on day 1 after the first immunization and replicated. This phenomenon could be explained by the fact that the tumor microenvironment is an immunoprivileged site, which provides protection against the host immune system (Yu et al., 2004; Loeffler et al., 2009). In studies by Yang et al. found that an LM-based vaccine could stimulate CD8^+^ T cells from tumor infiltrating lymphocytes (TILs) and suppress the recruitment and differentiation of Tregs in the tumor microenvironment (Yang et al., 2014). We also observed by immunohistochemistry and flow cytometry that our LM-based vaccine induced the accumulation of T cells within the tumor microenvironment. Based on the above results, we speculated that LM-based vaccines may deliver foreign antigens to tumor tissues in a targeted manner, and induce antigen-specific CTLs from TILs to participate in the antitumor immune responses at local tumor sites, thus contributing to tumor regression.

Ca^2+^ ion and ROS play a key role in both the initiation and effectuation of cell death (Rodriguez-Serrano et al., 2012; Nickel et al., 2016). Previously, Kim et al. reported that infection of tumor cells by an attenuated LM construct resulted in tumor cell death by activation of NAPDH oxidase and elevated levels of cytosolic ROS *in vitro* (Kim et al., 2009). We found that tumor cells from tumor-bearing mice immunized with rLM strains (LM4Δ*hly*::E7 and LM4Δ*hly*::*esat-6*) led to non-apoptotic cell death, the generation of intracellular ROS and elevated Ca^2+^ concentrations *in vivo*. Increases in intracellular ROS and Ca^2+^ levels were strongly associated with tumor cell death induced by LM-based vaccines. However, such tumor cell death induced by rLM strains was not influenced by delivery of specific antigen. Hence, these results indicated that LM4Δ*hly*::E7 can induce antitumor efficacy via non-specific direct killing of tumor cells.

LLO is necessary for intracellular proliferation of *Listeria*, which is a novel adjuvant with PAMP-like activity (Birmingham et al., 2008; Neeson et al., 2008). A 19-amino-acid PEST-like sequence (KENSISSMAPPASPPASPK) of the LLO signal peptide correlates with the virulence of *L. monocytogenes* (Decatur and Portnoy, 2000). Previous studies in our laboratory demonstrated that 14 amino acids (SMAPPASPPASP) of the PEST-like sequence were essential for the pathogenicity of *L. monocytogenes*, and showed similar activity. In this study, we fused the E7 gene with *hly* ORF in a 42 bp deletion region. LM4Δ*hly*::E7 was highly attenuated, as indicated by the reduced LD_50_ of this strain, and replicated only to a limited extent without causing severe pathology (Figure S2). However, our macrophage infection results showed that LM4Δ*hly*::E7 could infect and grow intracellularly in RAW264.7 macrophages despite the deletion of the LLO partial sequence, a modification which is necessary for successful delivery and presentation of foreign antigen. These observations showed LM4Δ*hly*::E7 to be less virulent while retaining its ability to present delivered antigen to the immune system. Also, the recombinant strain was stable and retained the expression of the LLO-E7 fusion protein after 30 passages *in vitro*, confirmed by RT-PCR (data not shown). Therefore, these properties suggest potential future clinical applications for this vector.

In summary, although TC-1-induced tumors gradually grew along with the expansion of Treg suppressed cells, immunization with LM4Δ*hly*::E7 successfully induced a robust cellular immunity. LM4Δ*hly*::E7 effectively retards tumor growth in a murine model of cervical cancer. Furthermore, we observed that the antitumor mechanisms were dependent on antigen-specific CTL activity and direct killing of tumor cells. Future studies include evaluation of LM4Δ*hly*::E7 as a candidate vaccine in a transgenic mouse model, in which immunological tolerance can be addressed. Furthermore, immunomodulatory agents such as Toll-like receptor 9 (TLR9) ligands and/or radiation therapy can be combined to further enhance their antitumor efficacy for application of LM-based vaccines in clinic. Taken in its entirety, our study provides strong evidence for future clinical translation of LM-based vaccines, as well as a basis for investigation into other HPV-related cancer models.

## Ethics statement

The mice were housed, handled and immunized at the animal biosafety facilities and all procedures were approved by the institutional animal experimental committee of Yangzhou University.

## Author contributions

YJ, YY, and XJ designed the experiments. WT and FD performed the experiments and analyzed the data. YY, ZP, XC, and XJ contributed reagents/materials/analysis tools. YJ, YY, and XJ wrote and revised the paper.

### Conflict of interest statement

The authors declare that the research was conducted in the absence of any commercial or financial relationships that could be construed as a potential conflict of interest.

## References

[B1] Azizoglu R. O. Elhanafi D. Kathariou S. (2014). Mutant construction and integration vector-mediated gene complementation in *Listeria monocytogenes*. Methods Mol. Biol. 1157, 201–211. 10.1007/978-1-4939-0703-8_17 24792560

[B2] Birmingham C. L. Canadien V. Kaniuk N. A. Steinberg B. E. Higgins D. E. Brumell J. H. (2008). Listeriolysin O allows *Listeria monocytogenes* replication in macrophage vacuoles. Nature 451, 350–354. 10.1038/nature06479 18202661

[B3] Chen Z. Ozbun L. Chong N. Wallecha A. Berzofsky J. A. Khleif S. N. (2014). Episomal expression of truncated listeriolysin O in LmddA-LLO-E7 vaccine enhances antitumor efficacy by preferentially inducing expansions of CD4+FoxP3- and CD8+ T cells. Cancer Immunol. Res. 2, 911–922. 10.1158/2326-6066.CIR-13-0197 24872025 PMC4160031

[B4] Coles R. M. Mueller S. N. Heath W. R. Carbone F. R. Brooks A. G. (2002). Progression of armed CTL from draining lymph node to spleen shortly after localized infection with Herpes Simplex Virus 1. J. Immunol. 168, 834–838. 10.4049/jimmunol.168.2.834 11777979

[B5] Cory L. Chu C. (2014). ADXS-HPV: a therapeutic Listeria vaccination targeting cervical cancers expressing the HPV E7 antigen. Hum. Vaccin. Immunother. 10, 3190–3195. 10.4161/hv.34378 25483687 PMC4514130

[B6] Decatur A. L. Portnoy D. A. (2000). A PEST-like sequence in listeriolysin O essential for *Listeria monocytogenes* pathogenicity. Science 290, 992–995. 10.1126/science.290.5493.992 11062133

[B7] Depuydt C. E. Thys S. Beert J. Jonckheere J. Salembier G. Bogers J. J. (2016). Linear viral load increase of a single HPV-type in women with multiple HPV infections predicts progression to cervical cancer. Int. J. Cancer 139, 2021–2032. 10.1002/ijc.30238 27339821

[B8] Ferlay J. Soerjomataram I. Dikshit R. Eser S. Mathers C. Rebelo M.. (2015). Cancer incidence and mortality worldwide: sources, methods and major patterns in GLOBOCAN 2012. Int. J. Cancer 136, E359–E386. 10.1002/ijc.29210 25220842

[B9] Grabowska A. K. Kaufmann A. M. Riemer A. B. (2015). Identification of promiscuous HPV16-derived T helper cell epitopes for therapeutic HPV vaccine design. Int. J. Cancer 136, 212–224. 10.1002/ijc.28968 24824905

[B10] Jia Y. Yin Y. Duan F. Fu H. Hu M. Gao Y.. (2012). Prophylactic and therapeutic efficacy of an attenuated *Listeria monocytogenes*-based vaccine delivering HPV16 E7 in a mouse model. Int. J. Mol. Med. 30, 1335–1342. 10.3892/ijmm.2012.1136 23027427

[B11] Killock D. (2015). Therapeutic HPV vaccine holds promise. Nat. Rev. Clin. Oncol. 12:686. 10.1038/nrclinonc.2015.180 26462124

[B12] Kim S. H. Castro F. Paterson Y. Gravekamp C. (2009). High efficacy of a Listeria-based vaccine against metastatic breast cancer reveals a dual mode of action. Cancer Res. 69, 5860–5866. 10.1158/0008-5472.CAN-08-4855 19584282 PMC3127451

[B13] Lee S. Y. Kang T. H. Knoff J. Huang Z. Soong R. S. Alvarez R. D.. (2013). Intratumoral injection of therapeutic HPV vaccinia vaccine following cisplatin enhances HPV-specific antitumor effects. Cancer Immunol. Immunother. 62, 1175–1185. 10.1007/s00262-013-1421-y 23615841 PMC3690484

[B14] Loeffler M. Le'Negrate G. Krajewska M. Reed J. C. (2009). *Salmonella typhimurium* engineered to produce CCL21 inhibit tumor growth. Cancer Immunol. Immunother. 58, 769–775. 10.1007/s00262-008-0555-9 18633610 PMC11030637

[B15] Mandai M. Hamanishi J. Abiko K. Matsumura N. Baba T. Konishi I. (2016). Dual faces of IFN-γ in cancer progression: a role of PD-L1 induction in the determination of pro- and anti-tumor immunity. Clin. Cancer Res. 22, 2329–2334. 10.1158/1078-0432.CCR-16-0224 27016309

[B16] Neeson P. Pan Z. K. Paterson Y. (2008). Listeriolysin O is an improved protein carrier for lymphoma immunoglobulin idiotype and provides systemic protection against 38C13 lymphoma. Cancer Immunol. Immunother. 57, 493–505. 10.1007/s00262-007-0388-y 17876582 PMC11030947

[B17] Nickel N. Cleven A. Enders V. Lisak D. Schneider L. Methner A. (2016). Androgen-inducible gene 1 increases the ER Ca^2+^ content and cell death susceptibility against oxidative stress. Gene 586, 62–68. 10.1016/j.gene.2016.03.055 27040980

[B18] Peng X. H. Hussain S. F. Paterson Y. (2004). The ability of two *Listeria monocytogenes* vaccines targeting human papillomavirus-16 E7 to induce an antitumor response correlates with myeloid dendritic cell function. J. Immunol. 172, 6030–6038. 10.4049/jimmunol.172.10.6030 15128786

[B19] Pillich H. Chakraborty T. Mraheil M. A. (2015). Cell-autonomous responses in *Listeria monocytogenes* infection. Future Microbiol. 10, 583–597. 10.2217/fmb.15.4 25865195

[B20] Qian Y. Zhang N. Jiang P. Chen S. Chu S. Hamze F.. (2012). Inhibitory effect of live-attenuated *Listeria monocytogenes*-based vaccines expressing MIA gene on malignant melanoma. J. Huazhong Univ. Sci. Technol. Med. Sci. 32, 591–597. 10.1007/s11596-012-1002-x 22886976

[B21] Rodriguez-Serrano M. Barany I. Prem D. Coronado M. J. Risueno M. C. Testillano P. S. (2012). NO, ROS, and cell death associated with caspase-like activity increase in stress-induced microspore embryogenesis of barley. J. Exp. Bot. 63, 2007–2024. 10.1093/jxb/err400 22197894 PMC3295391

[B22] Rothman J. Paterson Y. (2013). Live-attenuated Listeria-based immunotherapy. Expert Rev. Vaccines 12, 493–504. 10.1586/erv.13.34 23659298

[B23] Shahabi V. Reyes-Reyes M. Wallecha A. Rivera S. Paterson Y. Maciag P. (2008). Development of a *Listeria monocytogenes* based vaccine against prostate cancer. Cancer Immunol. Immunother. 57, 1301–1313. 10.1007/s00262-008-0463-z 18273616 PMC11030952

[B24] Singh R. Dominiecki M. E. Jaffee E. M. Paterson Y. (2005). Fusion to Listeriolysin, O., and delivery by *Listeria monocytogenes* enhances the immunogenicity of HER-2/neu and reveals subdominant epitopes in the FVB/N mouse. J. Immunol. 175, 3663–3673. 10.4049/jimmunol.175.6.3663 16148111

[B25] Stanley M. (2016). Preventing cervical cancer and genital warts - How much protection is enough for HPV vaccines? J. Infect. 72(Suppl.), S23–S28. 10.1016/j.jinf.2016.04.018 27211079

[B26] Torres-Poveda K. Burguete-Garcia A. I. Bahena-Roman M. Mendez-Martinez R. Zurita-Diaz M. A. Lopez-Estrada G.. (2016). Risk allelic load in Th2 and Th3 cytokines genes as biomarker of susceptibility to HPV-16 positive cervical cancer: a case control study. BMC Cancer 16:330. 10.1186/s12885-016-2364-4 27220278 PMC4879749

[B27] Wallecha A. Wood L. Pan Z. K. Maciag P. C. Shahabi V. Paterson Y. (2013). *Listeria monocytogenes*-derived listeriolysin O has pathogen-associated molecular pattern-like properties independent of its hemolytic ability. Clin. Vaccine Immunol. 20, 77–84. 10.1128/CVI.00488-12 23136118 PMC3535771

[B28] Wan X. Cheng C. Lin Z. Jiang R. Zhao W. Yan X.. (2015). The attenuated hepatocellular carcinoma-specific Listeria vaccine Lmdd-MPFG prevents tumor occurrence through immune regulation of dendritic cells. Oncotarget 6, 8822–8838. 10.18632/oncotarget.3558 25826093 PMC4496186

[B29] Wood L. M. Paterson Y. (2014). Attenuated *Listeria monocytogenes*: a powerful and versatile vector for the future of tumor immunotherapy. Front. Cell. Infect. Microbiol. 4:51. 10.3389/fcimb.2014.00051 24860789 PMC4026700

[B30] Yang A. Farmer E. Wu T. C. Hung C. F. (2016). Perspectives for therapeutic HPV vaccine development. J. Biomed. Sci. 23:75. 10.1186/s12929-016-0293-9 27809842 PMC5096309

[B31] Yang M. C. Yang A. Qiu J. Yang B. He L. Tsai Y. C.. (2016). Buccal injection of synthetic HPV long peptide vaccine induces local and systemic antigen-specific CD8+ T-cell immune responses and antitumor effects without adjuvant. Cell Biosci. 6:17. 10.1186/s13578-016-0083-9 26949512 PMC4778350

[B32] Yang Y. Hou J. Lin Z. Zhuo H. Chen D. Zhang X.. (2014). Attenuated *Listeria monocytogenes* as a cancer vaccine vector for the delivery of CD24, a biomarker for hepatic cancer stem cells. Cell. Mol. Immunol. 11, 184–196. 10.1038/cmi.2013.64 24488178 PMC4003383

[B33] Yu Y. A. Shabahang S. Timiryasova T. M. Zhang Q. Beltz R. Gentschev I.. (2004). Visualization of tumors and metastases in live animals with bacteria and vaccinia virus encoding light-emitting proteins. Nat. Biotechnol. 22, 313–320. 10.1038/nbt937 14990953

[B34] Yuji K. Nakada H. (2016). Compensation programs after withdrawal of the recommendation for HPV vaccine in Japan. Hum. Vaccin. Immunother. 12, 1321–1324. 10.1080/21645515.2015.1107686 26513303 PMC4963045

