# Supplementary material for: A Genetically Modified attenuated Listeria Vaccine Expressing HPV16 E7 Kill Tumor Cells in Direct and Antigen-Specific Manner
Source: Front Cell Infect Microbiol. 2017 Jun 29;7:279. doi: 10.3389/fcimb.2017.00279 (PMC5489629; doi:10.3389/fcimb.2017.00279)
Supplement: Supplementary file 1 [file Presentation1.PDF]

Figure S1 Intracelluar survival of recombinant strains and their parent strains in macrophages

RAW264.7 cells cultured in 24-well plates were infected with LM4(pNF8) or LM4Δ*hly*::E7(pNF8) at a multiplicity of infection of 1:20 for 1 h. The cells were incubated in medium containing 50 µg/mL of gentamicin for 1 h at 37°C to eliminate extracellular bacteria. Monolayers were washed twice and bacteria were collected following lysis with 0.1% Triton-X-100. The bacteria were titrated in Brain-heart infusion agar and CFUs were measured after 1 h and 5 h infection in the experiment.

Figure S2 Infection kinetics in organs of mice immunized with LM4Δ*hly*::E7 and LM4ΔactA/plcB.

C57BL/6 mice were intraperitoneally administered with 0.1 50% lethal dose (LD_{50}) of LM4Δ*hly*::E7 and LM4ΔactA/plcB(LM4Δ*hly*::E7,3.8×10^8 CFU, LM4ΔactA/plcB 5×10^7 CFU). The spleens and livers were homogenized on days 1, 2, 3 and 5 post-immunization. The bacterial numbers were determined by plating the cell suspensions on BHI agar.
