# Supplementary figures and images for: A Genetically Modified attenuated Listeria Vaccine Expressing HPV16 E7 Kill Tumor Cells in Direct and Antigen-Specific Manner
Source: Front Cell Infect Microbiol. 2017 Jun 29;7:279. doi: 10.3389/fcimb.2017.00279 (PMC5489629; doi:10.3389/fcimb.2017.00279)

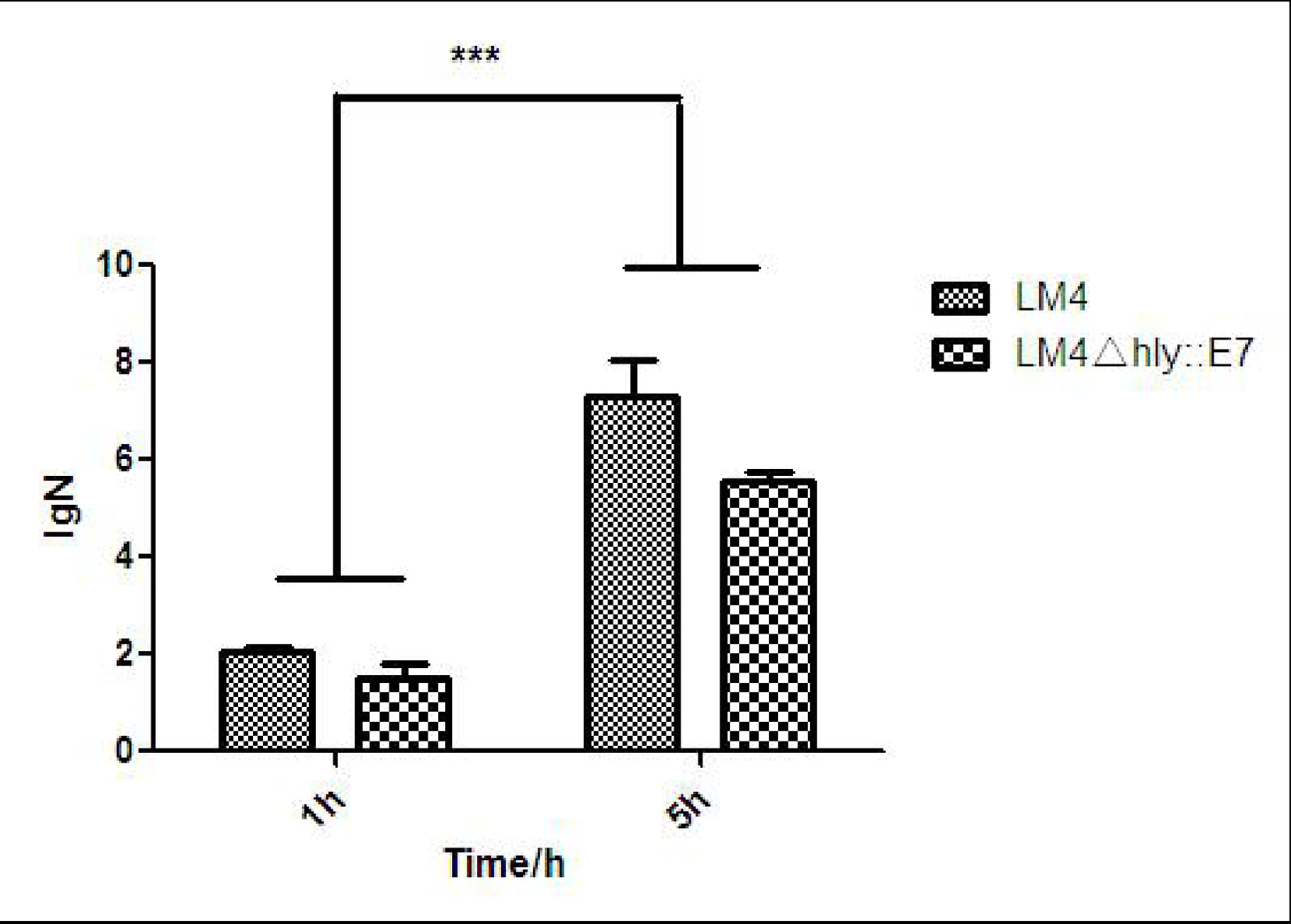

Supplement: Supplementary file 2 [file Image1.TIF]

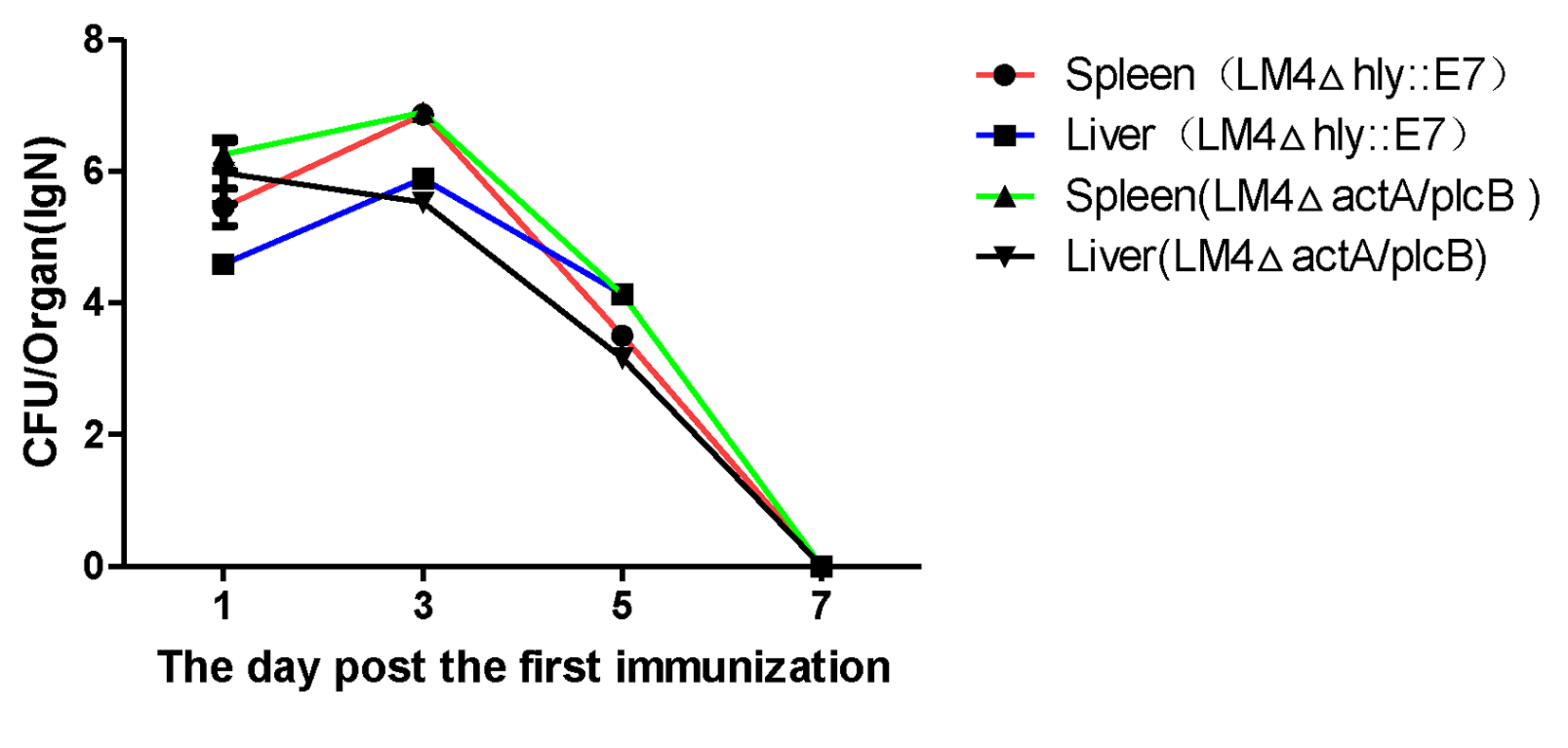

Supplement: Supplementary file 3 [file Image2.tif]
